# A Consensus-Based Grouping Algorithm for Multi-agent Cooperative Task Allocation with Complex Requirements

**DOI:** 10.1007/s12559-014-9265-0

**Published:** 2014-04-30

**Authors:** Simon Hunt, Qinggang Meng, Chris Hinde, Tingwen Huang

**Affiliations:** 1 Department of Computer Science, Loughborough University, Loughborough, UK; 2 Texas A&M University at Qatar, Doha, Qatar

**Keywords:** Consensus, Task allocation, Cooperation, Cognitive behaviours, Eusocial animals

## Abstract

This paper looks at consensus algorithms for agent cooperation with unmanned aerial vehicles. The foundation is the consensus-based bundle algorithm, which is extended to allow multi-agent tasks requiring agents to cooperate in completing individual tasks. Inspiration is taken from the cognitive behaviours of eusocial animals for cooperation and improved assignments. Using the behaviours observed in bees and ants inspires decentralised algorithms for groups of agents to adapt to changing task demand. Further extensions are provided to improve task complexity handling by the agents with added equipment requirements and task dependencies. We address the problems of handling these challenges and improve the efficiency of the algorithm for these requirements, whilst decreasing the communication cost with a new data structure. The proposed algorithm converges to a conflict-free, feasible solution of which previous algorithms are unable to account for. Furthermore, the algorithm takes into account heterogeneous agents, deadlocking and a method to store assignments for a dynamical environment. Simulation results demonstrate reduced data usage and communication time to come to a consensus on multi-agent tasks.

## Introduction

The rise in the use of unmanned aerial vehicles (UAVs) is becoming prevalent throughout the world. UAVs are finding valuable usage in performing military tasks that fall into the categories of the dull, dirty and dangerous [1]. As research develops, the future of UAVs looks progressively towards civilian activities [2]. Common applications include surveillance of power lines or pipes [3], disaster monitoring [4] and search and rescue operations [5]. As the applications for UAVs grow, so too does their need to cooperate to perform larger and increasingly complex tasks.

Creating a UAV to cover all situations and problems is difficult due to hardware and software limitations [6], and it becomes far easier to specialise UAVs to a precise problem. However, this reduces the UAV’s ability to solve a wide variety of tasks in a dynamic environment. With a diverse selection of UAVs that can form teams and work together to complete tasks, limitations of any single UAV can be solved. Using multiple UAVs will improve the efficiency with which a number of tasks can be performed by completing tasks in parallel. In this way then, a system that allows heterogeneous agents to assign and complete tasks together increases the flexibility of the system, an aspect of producing higher autonomy [7].

Of particular interest within the area of UAV, cooperation is the task assignment problem (TAP) which assigns a finite number of agents to complete a finite number of tasks as efficiently as possible. This problem can be solved with a centralised or decentralised solution, but current research looks at decentralised methods that provide a feasible solution for real-world application. Many researchers have solved the TAP using auction algorithms [8–10], where agents make bids for tasks and receive assignments based on their bids by a single auctioneer. Whilst task allocation for an individual agent is relatively simple, the difficulty occurs when a decentralised algorithm is used for consensus between all agents. One such solution that makes use of auction algorithms is the consensus-based auction algorithm (CBAA) [11], which solves the TAP for single-agent tasks that are defined as tasks that require a single agent to complete. The CBAA lets agents make bids for tasks and provides a system for decentralised consensus on assignments, giving us a conflict-free solution that has superior convergence and performance than other auction algorithms.

The consensus-based bundle algorithm (CBBA) [11] was created to solve an extension of the TAP where agents queue up tasks they will complete: individual agents take available tasks and compute every permutation given their current queue of tasks or “bundle”, where the highest rewarded permutation becomes their bid for that task. In this way, agents continually remove and revise new tasks as other agents find they can create a more valuable sequence with that task. Thus, the CBBA gives a conflict-free solution with a guaranteed 50 % optimality to the multi-assignment problem.

Extensions of the problem can be developed that simulate realistic situations by designing complex tasks with stricter requirements. The consensus algorithm needs to be developed to handle these new tasks, including tighter task selection and higher cooperative decision-making. The requirements that are looked at are multi-agent, equipment requirements and task dependencies, where a multi-agent task is defined as one that requires more than one agent to complete, an example of which would be using two UAV’s to carry construction material [12]. A task that requires specific equipment would require unique agents; Merino et al. [13] looked at using multiple heterogeneous agents for cooperative fire detection. Task dependencies are defined as tasks that require other tasks to be completed before they can start, creating a list of tasks that must be completed in order.

Two solutions for the multi-agent task allocation problem [14, 15] both have their limitations that make them unsuitable. Firstly, the creators of the CBBA extended their algorithm for heterogeneous cooperation [14]; this extension solved duo cooperation constraints where a simulation would contain two agent types that solve three different types of tasks. Solo tasks required one type of agent; preferred duo tasks scored greater for the assignment of two different agents and required duo tasks needed one of each agent type. But this solution is limited to two agents, and the proposed solution here will allow any number of agent requirements on tasks. Secondly, another solution to the multi-agent problem [15] used a central solver to group-related tasks into a set and assign enough agents to complete them. But using a centralised algorithm will not provide a robust and feasible solution for real-world applications. This paper provides a solution for the decentralised assignment of multiple tasks that require any number of agents for their completion. Solving this problem can increase the cooperation of UAVs to an improved autonomous operational level further reducing the need for human interaction. To achieve this, agents need to develop an increased awareness of what other agents are planning more so than required for the CBBA. Agents must plan their own schedules around that of others and come to complex agreements on task order. As the complexity of decision-making increases so too does the requirement for information needed to make a decision and the underlying communication required [16]. The reliance on decisions of other agents adds to the problem, to deal with this challenge, inspiration can be drawn from the cognitive behaviours of eusocial animals using their complex behaviours for group decision-making [17–19].

Using the framework set up by the CBBA, we extend the algorithm to account for existing limitations, leading us to the consensus-based grouping algorithm (CBGA).

## Cognitive Decision-Making from Eusocial Animals

Eusocial animals, like the majority of ant species, a number of bee species and a few wasp species have some similarities to that of robotic cooperative systems [17, 20, 21]. Unlike most animal species, eusocial animals focus on the group rather than the individual. An ant, for instance, has evolved to put the success of the colony ahead of itself; ants have been shown to use self-sacrificial defences to protect the nest [22]. Similarly with a cooperative system, an individual agent should focus on maximising the performance of the group as a whole rather than its own performance. Multi-agent tasks present a unique set of problems relating to team organisation and cooperation. The CBBA as the bases for this extension is focused entirely on the individual and improving its score which in turn improves the overall team score. With multi-agent tasks, a greater individual improvement is not necessarily the best improvement for the team where incomplete team assignments give no reward. Taking inspiration from collective animal behaviours of large groups such as ants can be useful in developing algorithms for group decision-making. Ant nests allocate specific workers to specific tasks without any central or hierarchical control [23, 24]. Whilst the task allocation is individual centric and the decision is made by an individual, it must still be beneficial to the group. Some decisions will reduce an agent’s contribution but overall increase the team’s performance, the allocation algorithm must account for both loss of time and score by not fully allocating multi-agent tasks.

Bees perform task partitioning where a task is split up into a number of steps that are performed by multiple bees [25]. This focuses the hive on the task and its division rather than the individual performing the task. As part of a “hygienic behaviour”, worker bees remove diseased brood cells from the hive. This requires two operations, the removal of the cap on the cell followed by removing the diseased brood. Often, an individual worker bee will focus on either uncapping or removal. Bee colonies show complex cooperative behaviours for the self-organisation and allocation of workers in the hive [26]. Multiple systems have been proposed that show how bee colonies come to collective decisions in tasks such as the allocation of workers on nectar sources with changing environmental conditions [27–29]. Detrain and Deneubourg [30] show how if–then rules embedded in ant behaviours, however simple in their logic ultimately produce efficient group-level responses for objectives such as resource acquisition and risk avoidance. Further that these behavioural rules coupled with self-organising processes provide a robust and efficient method for problem solving. A difficulty encountered with multi-agent tasks is that agents can get stuck assigned to tasks that no other agents plan to assist with. When a multi-agent task has insufficient assignments, the task cannot be completed and will not score. Bees have been shown to exhibit behaviours that result in a form of task quitting by becoming insensitive to certain stimuli for a period of time [31]. This process allows bees to reassign to high-priority areas and would help improve agent assignments by distributing agents to tasks with a higher demand. With the inclusion of multi-agent tasks, the developed algorithm has a greater focus on the assignments and score of other agents. Using the task quitting method from bee colonies and the team-focused assignments of ant nests improvements in the cooperative assignment for multi-agent tasks can be compared.

## Consensus-Based Algorithms

### Consensus-Based Auction Algorithm

The consensus-based auction algorithm (CBAA) solves single assignment problems using both auction and consensus in a decentralised system [11]. The algorithm contains two phases that alternate until assignments and consensus are achieved. The first phase of the algorithm is the auction process, whilst the second is a consensus algorithm that is used to converge on a winning solution. The CBAA by iterating between the two phases can exploit the benefits of both auction and consensus algorithms. Robustness and computational efficiency are achieved from the auction algorithm whilst the decentralised consensus algorithm can exploit network flexibility and converge on a conflict-free solution. The CBAA was shown to provide a conflict-free, feasible solution, which previous algorithms were unable to account for.

#### Phase 1: The Auction Process

The first phase of the algorithm is the auction process. Here, each agent *i* places a bid for a task *j* asynchronously with all other agents. Every agent stores and updates two vectors *x* and *y* of length *N*_*m*_ where *N*_*m*_ is the number of tasks in the simulation, both are initialized as zero vectors. The first vector *x*_*i*_ records the task list for agent *i*, if agent *i* has been assigned to task *j*, then $x_{ij} = 1$, and 0 if not. The second vector is the winning bids list *y*_*i*_ which stores the current highest winning bid for each task that agent *i* has knowledge of. Agent *i* calculates the cost *c*_*ij*_ for each task *j*, subtracting cost from a fixed reward to produce a task score $S_{i}^{j}$. Agent *i* then places a bid that is greater than any current bid for a task that provides the maximum individual increase in score.

#### Phase 2: The Consensus Process

The second phase of the CBAA is the consensus section of the algorithm, which involves communicating the winning bid lists of each agent and coming to a consensus on assignments. Here, agents converge on a conflict-free solution using a consensus strategy that converges on a list of winning bids.

Each agent communicates their winning bid list to all other agents within communication range. *G*(*τ*) is a symmetrical adjacency matrix used to determine whether there is a communication link between agents. If there exists a link between agents *i* and *k* at time *τ*, then $g_{ik} (\tau ) = 1$, otherwise it is 0. When such a link exists, agents are said to be neighbours. At an iteration of phase 2, agent *i* sends its winning bid list *y*_*i*_ to all its neighbours. Likewise, agent *i* receives a winning bid list *y*_*k*_ from each neighbour. Consensus is performed on the winning bid lists such that agent *i* replaces *y*_*ij*_ values with the largest value between itself and its neighbours. If an agent finds that a task it had selected has been outbid, then it will lose that assignment.

### The Consensus-Based Bundle Algorithm

The major downside to the CBAA is that whilst at a specific time each agent can select the most optimal task to complete, it does not take into account future selections. When a number of tasks are located in close proximity, a single agent can perform all the tasks rather than sending an agent for each task. Researchers addressed the problem by grouping assignments into bundles for bidding [14, 24–27] creating the multi-assignment problem, where each agent bids for multiple tasks. Each assignment combination or bundle was treated as a single item for bidding which led to complicated winner selection methods. The CBAA was extended to the multi-assignment problem developing the CBBA [11], which gives a conflict-free solution with a guaranteed 50 % optimality. In the CBBA, each agent has a list of tasks potentially assigned to it, but the auction process is done at the task level rather than at the bundle level as previous algorithms had done. Similar to the CBAA, the CBBA contains two distinct phases for controlling the allocation and consensus of tasks.

#### Phase 1: The Bundle Construction

During the first phase, an agent internally builds up a single bundle containing all the tasks it plans to complete and updates it as the assignment process progresses. Each agent continually adds to its bundle until it is incapable of adding any other tasks. Agents carry two lists of tasks: the bundle *b*_*i*_ and a path *p*_*i*_. The bundle contains all tasks an agent will complete and is grouped in the order tasks were added, and the path, however, contains an ordered sequence of tasks that agent *i* will perform. Using $S_{i}^{{p_{i} }}$ as the total reward for agent *i* performing the tasks along the path *p*_*i*_ and where $S_{i}^{{p_{i} \theta_{n} \{ j\} }}$ is the total reward from inserting task *j* into position *n* of the path *p*_*i*_. If a task *j* is added to the bundle *b*_*i*_, it incurs the score improvement of

1 $$ c_{ij}[b_{i}] = \left\{ {\begin{array}{*{20}l}  {0}, \hfill &
 {\text{if }}j \in b_i \\ {\text{max}}_{n \le |p_{i}|}
S_{i}^{p_{i}\theta_{n}\{j\}} - S_{i}^{p_{i}},  \hfill &
{\text{otherwise}} \\  \end{array} ,}\right.  $$

where |·| denotes the cardinality of the list, and $\theta_{n}$ denotes the operation that inserts the second list right after the *n*th element of the final list. A new task is inserted into the current path at all possible locations to find the highest increase in reward. Each agent carries five vectors: a winning bid list *y*_*i*_, a winning agent list *z*_*i*_, an agent update time *s*_*i*_, a bundle *b*_*i*_ and the corresponding path *p*_*i*_. The winning agent list *z*_*i*_ stores the agent currently assigned to each task such that when *z*_*ij*_ = *k* agent *i* believes that agent *k* is assigned to task *j*. An agent needs to know not only if it is outbid on a task it selects but who is assigned to each task as well; this enables better assignments based on more sophisticated conflict resolution rules.

#### Phase 2: Conflict Resolution

Similar to the CBAA, the CBBA runs a consensus phase to remove agents bidding for the same task. In the CBAA, agents made bids on single tasks and released them upon receiving a higher value in the winning bids list. On the contrary, in CBBA, agents make bids on tasks based on their currently assigned task set. If an agent is outbid on a task, then the score values for all the following tasks are no longer valid. Therefore, when an agent is outbid, it must release all the tasks added after the outbid task.

When agent *i* receives a message from another agent, *k*, *z*_*i*_ and *s*_*i*_ are used to determine which agent’s information is the most up-to-date for each task. There are three possible actions agent *i* can take on task *j*:

1. $${\text{Update:}} \,y_{ij} = y_{kj} ,\quad z_{ij} = z_{kj}$$
2. $${\text{Reset:}} \,y_{ij} = 0,\quad z_{ij} = \emptyset$$
3. $${\text{Leave:}} \,y_{ij} = y_{ij} ,\quad z_{ij} = z_{ij}$$

Using a lookup table, an agent determines whether it should update, reset or leave the bid. Agents compare their knowledge on task *j* between the receiver *i* and the sender *k* along with when each agent last received communication from the agent they believe is assigned to task *j*. Agents alternate between the two phases until they converge on a conflict-free solution.

### Problem

The CBBA is limited to single-agent tasks and is unable to handle further restrictions on which agents can complete those tasks. This paper develops an algorithm that can deal with and provide a conflict-free solution to the following restraints.

- Tasks require 1 to *n* agents
- Tasks require specific equipment or sensors
- Tasks can have an order of completion

Agents will need to form groups containing the correct equipment before being able to complete a task. Additionally, tasks might require a specific order of completion.

#### Task Assignment Problem

The task assignment problem is a combinatorial optimisation problem that tries to find the least-cost solution between two disjoint sets. There is a set of agents $I \equiv \{ 1, \ldots ,N_{n} \}$ and a set of tasks $J \equiv \{ 1, \ldots ,N_{m} \}$. With a valid assignment, each agent $i \in I$ must be assigned to a task and each task $j \in J$ must have exactly one agent assigned.

An agent has a cost associated with it for completing a task. Let *c*_*ij*_ be the non-negative cost of assigning the *i*th agent to the *j*th task. The objective is to assign each task one agent in such a way as to minimise the overall cost of completing all the tasks. If we define a binary variable *X*_*ij*_ where *X*_*ij*_ = 1 to indicate agent *i* is assigned to task *j*, otherwise $X_{ij} = 0$. Then, the total cost of the assignment is equal to (2).

2 $$\sum X_{ij} *c_{ij} \quad {\text{for}}\quad i = 1 \;{\text{to}}\; n, \quad j = 1 \;{\text{to}}\; m.$$

For an assignment to be efficient, we say the task allocation must be valid and the cost is minimised (3).

3 $${\text{Cost}} = \hbox{min} \left( {\mathop \sum \nolimits X_{ij} * c_{ij} \quad {\text{for}}\quad i = 1 \;{\text{to}}\; n, \quad j = 1 \;{\text{to}}\; m} \right).$$

#### Restricted Task Assignment Problem

As we extend the TAP, we are creating restrictions that limit which agents are valid depending on their equipment but we remove the single assignment restriction. Each agent *i* can be assigned to multiple tasks as part of the CBBA; conversely, each task *j* can similarly have multiple agents assigned to it. Each task *j* contains an agent requirement *L*_*j*_ that specifies how many agents are required for the task. Although multiple agents can potentially be assigned to a single task, the cost function will stay the same; however, the algorithm will not limit *X*_*ij*_ = 1 to a single instance for each *j*. Instead for an assignment to be valid, $\mathop \sum \nolimits (X_{ij} \forall i) = L_{j}$ must be true. Additionally, there is a list of equipment $E \equiv \{ e_{1} ,e_{2} \ldots ,e_{Nq} \}$ found in the assignments where *e*_*i*_ is a list of equipment that agent *i* has such that $e_{i} \subseteq E$. Similarly, task *j* requires a specific list of equipment *e*_*j*_ where $e_{j} \subseteq E$. When $e_{i} \mathop \cap \nolimits e_{j} \ne \emptyset$, agent *i* can assist on task *j*. A successful assignment is worked out using

4 $$e_{j} \backslash \left\{ {e_{i} |X_{ij} > 0} \right\} \ne \emptyset \;{\text{and}}\; \left\{ {i |X_{ij} > 0} \right\} = L_{j},$$

where “\” is the set compliment, thus when (4) is true task *j* has been successfully assigned with the correct equipment and number of agents.

When making assignments with task planning from the CBBA, tasks are only available for bidding when all requirements have been met, agents should be able to plan all tasks in advance.

Therefore, we create a set of prerequisites $P_{j} \in J$ for each task *j* containing which tasks must be completed before the related task can be attempted. When $P_{j} = \emptyset$, task *j* has no prerequisites and availability is limited to the highest bidder as before. Using the existence of a winning bid *Y*_*ij*_, we can begin to establish whether a required task is going to be completed.

5 $$X_{ij} = \mathop \sum \nolimits (Y_{im} > 0)\;\forall \;m \in P_{j} .$$

when (5) is positive agent *i* can potentially complete task *j* where all prerequisite tasks *m* have complete assignments. Depending on the specific requirements of a task, we can add either type of restriction to determine whether a specific agent can complete a chosen task.

## Consensus-Based Group Algorithm

The consensus-based bundle algorithm (CBBA) was created to solve an extension of the TAP where agents are allowed to queue up tasks they will complete. Individual agents take available tasks and compute every permutation given their current queue of tasks. The greatest increase in reward is used as the tasks bid. Agents continually add and remove tasks as other agents find higher valued sequences with that task. However, the algorithm does not consider multi-agent requirements of the task, a multi-agent task is defined as a task that requires more than one agent for it to be completed. Further to that, current algorithms [14, 15, 32] simplify the problem by allocating agents into groups and solving as a regular TAP. These algorithms represent groups of agents as individual agents and solve the problem with the CBBA. The CBGA proposed in this research will solve the multi-agent task problem but keep agents independent allowing them to freely form groups to complete multi-agent tasks.

Further requirements are considered by placing equipment requirements onto each task. This restricts which agents can complete specific tasks and creates an algorithm that can handle heterogeneous agents. After developing a structure for assigning multiple agents to a single task, we can use cooperation to solve equipment limitations. Current algorithms are unable to account for the assignment and consensus when multiple agents are required for a single task. Using the framework set-up by the CBBA, the algorithm is extended to account for the new requirements. Tasks will require varying numbers of agents and equipment. Further additions to autonomy can be achieved by adding task scheduling to the framework where tasks are dependent on the completion of other tasks.

The CBBA provides a conflict-free solution with a guaranteed 50 % optimality; however, once we expand on the situation and increase the requirements of tasks the algorithm cannot complete these problems. The focus of this research is to extend the CBBA to manage the increased complexity of task requirements. These extensions lead to the CBGA [33].

### Local Data

With the CBBA, task and agent information are stored locally at the beginning of the simulation. Each agent stores two vectors, a winning bids list *y*_*i*_ and the winning agent list *z*_*i*_. When transferring this data storage system over to a multi-task multi-agent system, problems are caused with consistency between agents and tasks. Different tasks can have varying number of agents assigned to them, for tasks that require multiple assignments a vector cannot store data of each assignment. Therefore, with the CBGA, we need to modify the storage of these values to allow multiple agent assignments. Originally, the winning bids list *y*_*i*_ and the winning agent’s list *z*_*i*_ are two vectors of length *N*_*m*_ where *N*_*m*_ is the number of tasks in the algorithm. With these two vectors, each agent can keep track of the highest bid for each task with *y*_*i*_ and which agent made that bid with *z*_*is*_. Once we make tasks require multiple agents, we must convert both vectors into matrices to keep track of contribution bids and multiple winners. This gives two matrices of size $N_{m} *{ \hbox{max} }(L_{j} )$ where *L*_*j*_ is the required number of agents for task *j*. However, each task *j* that has an *L*_*j*_ less than the ${ \hbox{max} }(L_{j} )$ will leave unused space in the matrix. Additionally, we now need to communicate two matrices rather than two vectors.

Instead of using two matrices, we can remove redundancy by merging them into a single matrix *X* which contains all the winning bids and is of size $N_{n} *N_{m}$ where *N*_*n*_ is the number of agents in the simulation. This allows the algorithm to use rows to display tasks and columns to display agents, thus *x*_*ij*_ corresponds to the bid agent *i* has made for task *j* otherwise 0 if the agent has not made a bid.

Using the values in each row, we can determine the total score for completing a task as shown in (6).

6 $$C_{j} = \mathop \sum \limits_{i} x_{ij} .$$

where *C*_*j*_ is the score for completing task *j*. Further, the number of instances of nonzero values in each row should never exceed the number of required agents *L*_*j*_.

In a dynamic multi-agent system, we cannot assume each agent will store data in the same order, in a dynamic environment where agents look after their own data, new tasks or agents can be discovered in different orders to other agents. Therefore, we cannot use the matrix index of *X* as a reliable identifier for an agent or task. Agents therefore need to store individually a separate agent vector *I* that contains all known agent IDs and a task vector *J* that contains all known task IDs. These two vectors are used as lookup tables to the assignment matrix *X* where each vector can be ordered differently for each agent. With this new matrix shown in Table 1, agents can store data dynamically and build up a list of agent to task assignments as they discover new agents or tasks in the environment. When a new agent is discovered an extra, column is created in the assignment matrix and the ID is added to the agent vector. Agents can individually build up their assignment matrix in different orders but still communicate the data reliably without conflict using their own task and agent vectors. Update times from agents can continue to be stored in a vector *s*_*i*_ and are similarly identified using the agent vector *I*.

**Table 1:**
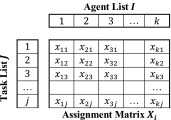
Dynamic variable storage for agent *i*, where *X*_*ijk*_ references the winning bid agent *i* believes agent *k* has made for task *j*

### Bundle Construction

In phase 1, each agent constructs a bundle of tasks *b*_*i*_ and the ordered path for those tasks *p*_*i*_. Bundle and path construction works as developed in the CBBA [11] but with new task restrictions. Each task provides a fixed score *r*_*j*_ for each agent as a reward according to the task requirement and an agent’s capability. The overall cost function for an agent *i* completing task *j* is worked out as

7 $$c_{ij} = r_{j} - (d_{ij} + t_{j} ),$$

where *d*_*ij*_ is the distance agent *i* is from task *j* and *t*_*j*_ is the time it takes to complete task *j*. The sum of these costs is taken away from the reward *r*_*j*_ of completing task *j*. The cost function is calculated using the agent’s current position, for pre-planning a path of tasks to complete the previous task location must be used. Calculating the current tasks, cost will vary depending on the previous task. An agent must maximise the reward finding the best fitting path for the tasks. When a task is placed inside the path, *d*_*ij*_ takes the form *d*_*lj*_ where *l* is the previous tasks location. The value *c*_*ij*_ is used to work out whether a bid will be successful against another agent. However, when working out the minimum cost in *p*_*i*_, we need to account for other agents involved in the task and their travel times, thus overall cost for task *j* is

8 $$c_{ij} = r_{j} - \left( {\hbox{max} \left( {d_{mj} \left( {\forall m \in M,X_{ij} > 0 } \right), d_{ij} } \right) + t_{j} } \right),$$

where $\forall m \in M,X_{ij} > 0$ finds the latest arrival time to task *j* out of the assigned agents in *X*_*ij*_ and agent *i*.

Adding together, all the costs for an agent’s path gives us $S_{i}^{{p_{i} }}$ the total score for agent *i* with path *p*. We can describe the score for slotting task *j* into position *n* as $S_{i}^{{p_{i} \theta_{n} \left\{ j \right\}}}$. When a task is updated with a new agent, the previous paths do not immediately need to be discarded; however, the path time may change depending on the new assignment.

A problem with the CBBA for multi-agent assignments is that agents decide which tasks to do based on their own individual improvement. This is a result caused by the multi-agent requirements where agents cannot receive a score from a multi-agent task that do not have all the required assignments. To create team-focused assignments, agents will calculate the total value for a successful task rather than just their individual contribution. This prioritises agents into completing team-based tasks that already have assignments despite it rewarding less for the individual [23].

**Fig. 1:**
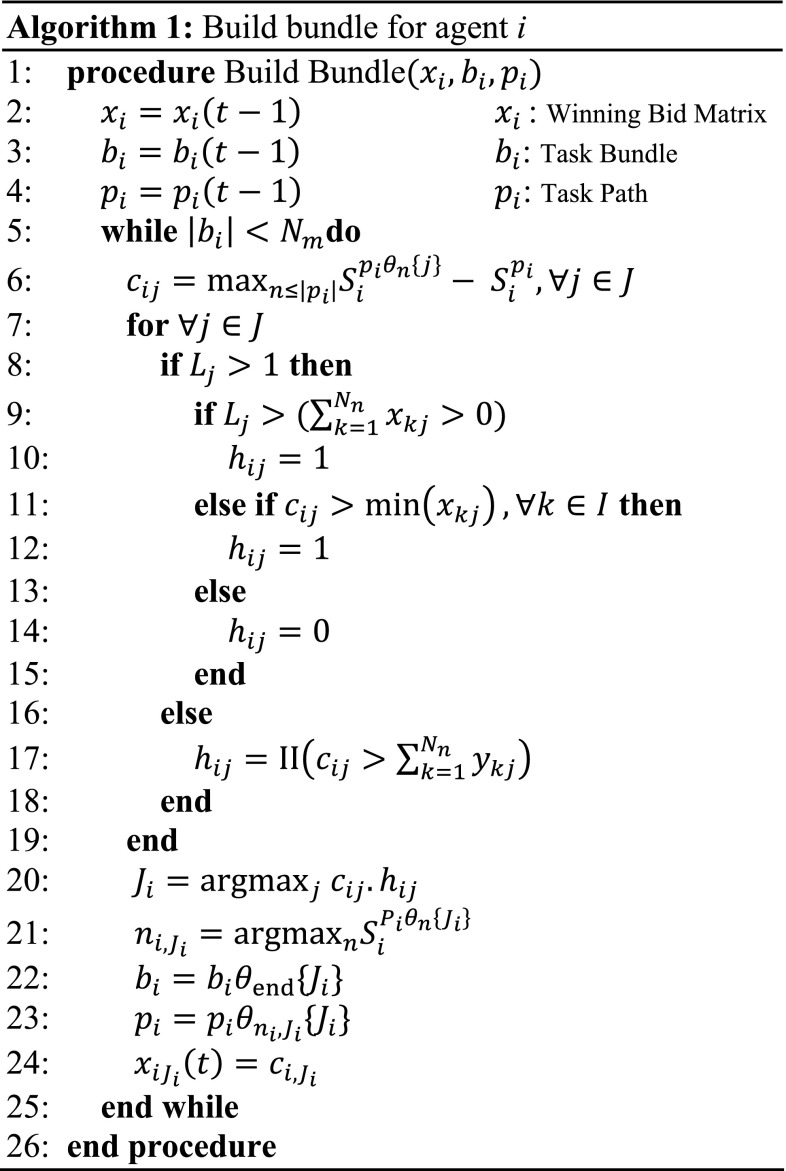
Bundle construction for the CBGA

The bundle algorithm shown in Fig. 1 is similar to that in the CBBA [11] but uses different costing functions, data storage and allows multiple assignments. The bidding aspect of the algorithm will not change with the complexity of tasks; however, the cost functions will change as the deciding factor in who should complete a task. However, assignments for multi-agent tasks will function differently to the CBBA. Multi-agent tasks are added to the valid task list *h*_*ij*_ when either the task is not full (line 9, Fig. 1) or the task is full but the agent has a higher bid than smallest current bid in the task (line 11, Fig. 1). Single-agent tasks are added to the valid task list *h*_*ij*_ in the same way as the CBBA (line 17, Fig. 1) where I(·) is the unity if the argument is true and zero otherwise. From the list of valid tasks *h*_*ij*_, the task that provides the greatest improvement in score at position *n*_*i*,*ji*_ in the path *p*_*i*_ is selected and added to the agent’s assignments. This process is repeated until the agent is unable to add any more tasks that improve its score.

### Consensus

**Fig. 2:**
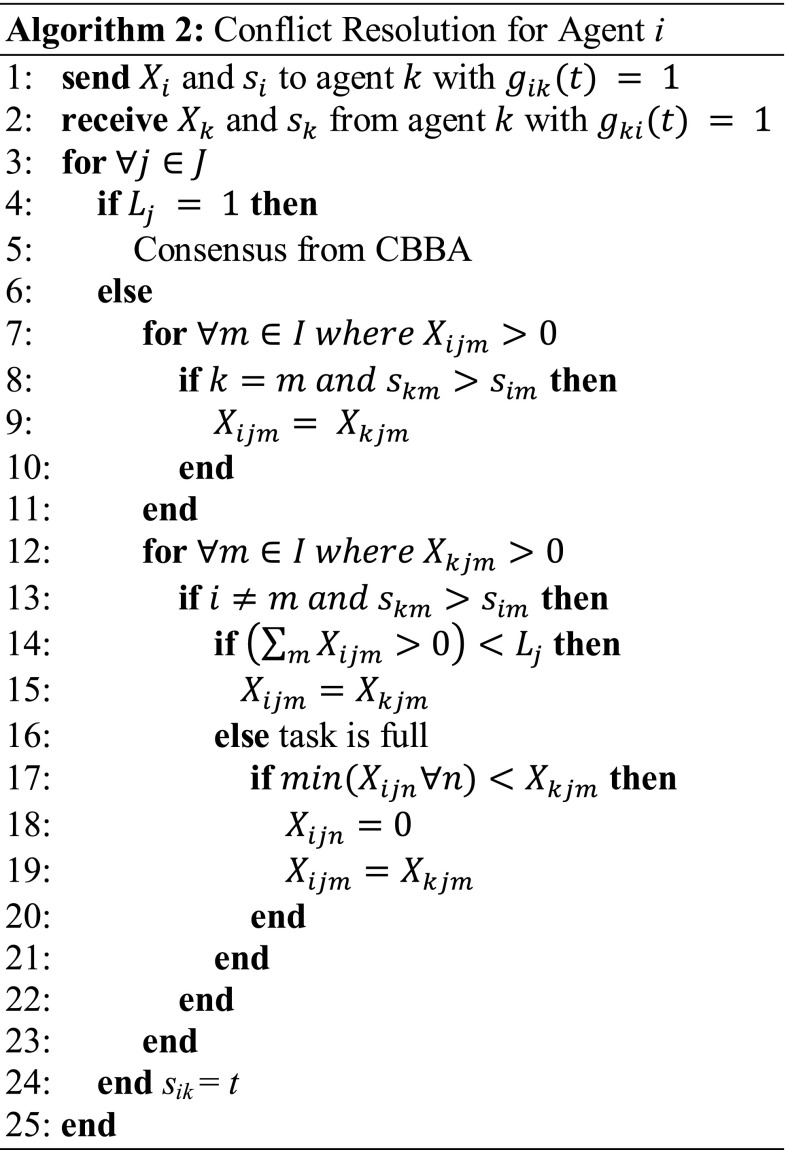
Conflict resolution for the CBGA for multi-agent tasks. Consensus performed between two agents *i* and *k* updating agent bid list for agent *i*

Phase 2 of the algorithm takes communications received from neighbours and analyses their knowledge of assignments to come to a consensus. Each agent communicates their winning bid matrix *X*_*i*_ and the time stamp *s*_*i*_ displaying the last information update from each of the other agents. As agents receive assignment data from neighbours, they will build up and store assignment matrixes for each neighbour where *X*_*ijm*_ is defined as the bid agent *i* thinks agent *m* has made for task *j*. The consensus algorithm is split into two sections, the first section (line 4–6, Fig. 2) deals with tasks that require a single agent, using *L*_*j*_ to determine the number of agents required for task *j*. Tasks requiring a single agent will require the same consensus algorithm as found in the CBBA [11]. The consensus algorithm assumes only valid bids are made during the bundle construction algorithm, thus no changes are required for single-agent tasks. This paper focuses on tasks that require more than one agent and thus require a different consensus algorithm to converge on an answer for the multi-agent tasks.

The second section (line 7–22, Fig. 2) contains the multi-agent consensus part of the CBGA, which is split into two phases: the first correlates the receiver’s current information with that of the sender and secondly, the receiver takes new information from the sender and merges it with its own data to produce a consistent set of agreed information. The CBBA used a table for determining whether to update, leave or reset information; with the extended problem, this becomes problematic. When another agent has differing assignments, it does not necessarily require leaving or updating the information as done in the CBBA, and the information could merge causing both agents to be correct. Further complications come when equipment requirements are taken into account. The algorithm is split into two phases to best handle the incoming information; by correcting each agents information, the agent can merge incoming data better by not having to account for mistakes in its own data.

The first phase (line 7–11, Fig. 2) takes all the winning assignments the receiver has stored and compares how correct that information is with the sender. Comparing *s*_*km*_ > *s*_*im*_ where *s*_*km*_ is the last time, the sender *k* had communication with *m*, the receiver *i* checks if it has had an earlier update with *m*. If the sender has had a better update time, then their data will be more accurate, and this could be either a better bid or that the agent is no longer assigned to the task. When $X_{ijm} > 0$, agent *i* believes an assignment is taking place between agent *m* and task *j.* If *s*_*km*_ > *s*_*im*_, then agent *k* has been updated by *m*; more recently, thus, its information has better reliability.

During the second phase (line 12–23, Fig. 2), the receiver’s information is updated with new information from the sender. From the first phase, an agent knows that all of its assignments, according to the sender, are currently up-to-date. Following this, the agent can proceed through each task and evaluate any additional data from the sender. When the sender *k* has an agent *m* assigned to task *j* that is neither the receiver nor assigned by *i* to the given task (line 12–13, Fig. 2), the algorithm can update the agents winning bid list with the new assignment.

When the algorithm replaces an agent in a current group, it must replace an agent that is carrying at least one piece of identical equipment, as the bidding process will not let a group fill up without meeting the required equipment list. If $\sum\nolimits_{m} {(X_{ijm} > 0) < L_{j} }$, then there is a space available in the current task, and if $e_{m} \in e_{j} \backslash \left\{ {e_{i} |X_{ij} > 0} \right\}$, there is still a requirement for an agent *m* with specific equipment *e*_*m*_ without the need to replace an assigned agent.

If there are not available spaces in the group, then

9 $$\left( {\hbox{min} \,X_{ijn}\, \forall n} \right) <\, X_{kjm} \;{\text{and}}\; e_{n} = e_{m},$$

is used to find, if feasible, an agent with the same equipment as *m* and with a lower contribution score. If these conditions are met, then the algorithm can replace the lower scored agent. To avoid any chance of deadlocking, we must account for the small chance of scores being tied. In this situation, the agent with the highest ID gets priority. It is a systematic way to guarantee a winner despite equal scores.

The purpose of a task quitting system is to move resources to higher-demand areas; in the case of multi-agent task assignment, task quitting will help remove agents from tasks with unmet requirements to assist in tasks that are closer to meeting the requirements.

10 $$\left( {\mathop \sum \limits_{i} X_{ij} > 0} \right) <\, L_{j} \;{\text{and}}\;t - T_{j} > \delta .$$

As agents assign tasks, they record the time of assignment *T*_*j*_. Using (10), when any agent finds a task they are assigned to that is not filled within the time threshold *δ*, they will remove and mark the task unfeasible until other agents assign themselves. With multi-agent tasks only providing a score for a complete assignment, this method of task quitting allows agents to adapt to the changing demand of tasks. When a task’s requirements are partially met, it is closer to scoring than an unassigned task, thus it is naturally in more demand to be completed. Inspired by the cognitive behaviours of worker bees in hives [31], this addition allows agents to prioritise completing partially full tasks.

## Performance

### Test Scenario

Each test contains 20 tasks with a varying number of agents where $N_{n} \in \{ 1, \ldots ,10\}$. The objective of each experiment is to maximise the total agent score. The overall score of each experiment is the sum of all rewards for the completed tasks minus the cost of distance travelled. Multi-agent tasks will reward a score to each agent involved signifying the difficulty and importance of such tasks. Observations will be made on the overall impact on the score and the amount of communications per consensus. Communication between agent *i* and agent *k* where allocation data are sent is counted as a single communication step. Each experiment was run 100 times for each increasing number of agents.

### Communication

**Fig. 3:**
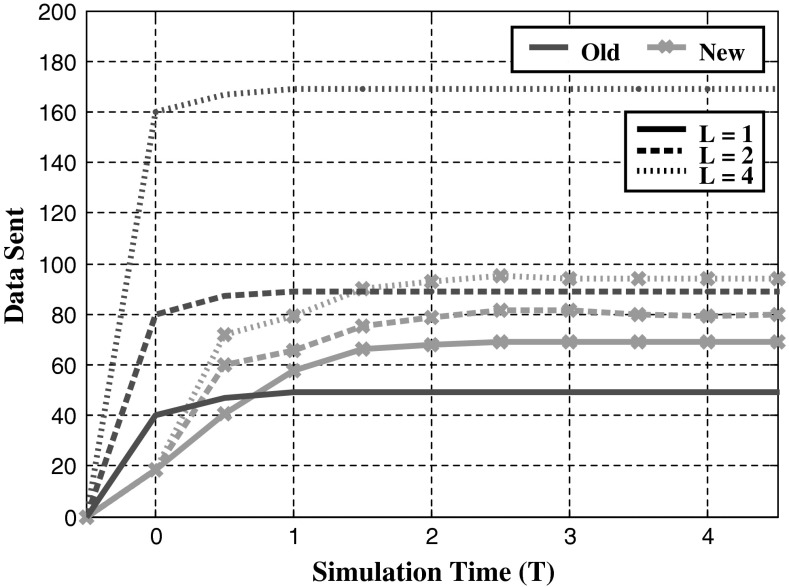
Average amount of data sent in progressive steps through a simulation. Simulated experiments contain 10 agents completing 20 tasks requiring *L* agents per task. Data are calculated as an individual piece of information sent from one agent. *Markers* (×) used to show the new data system

With changes made to the data structures on each agent, comparisons can be made between the two different data storage methods. Adapting the original method to multi-agent tasks uses multiple vectors to store each bid. The new method uses a dynamic matrix for each agent. Assignments are sent individually in the form $[i j x_{ij} ]$. Figure 3 shows that the new system reduces the amount of data sent for multi-agent tasks. Data sent with the new system gradually increases over the simulation. The old method involved sending the entire assignment data regardless of whether bids had been made. With the new system, redundant data are removed allowing agents to send only the required information.

### Multi-Agent Tasks

**Fig. 4:**
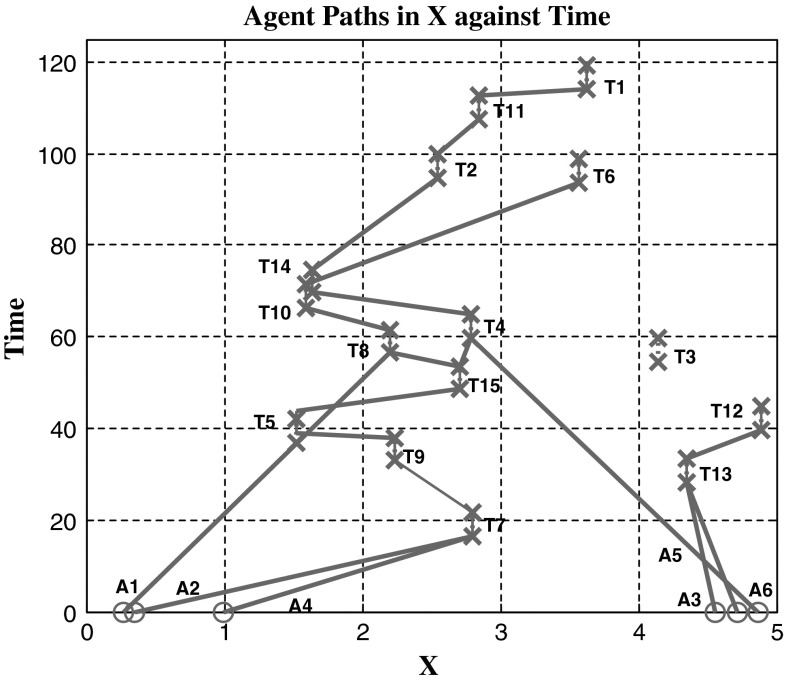
Cross-section of agents (*A*) movement through time and the *X* axis to complete tasks (*T*). Each task requires two agents for completion

**Fig. 5:**
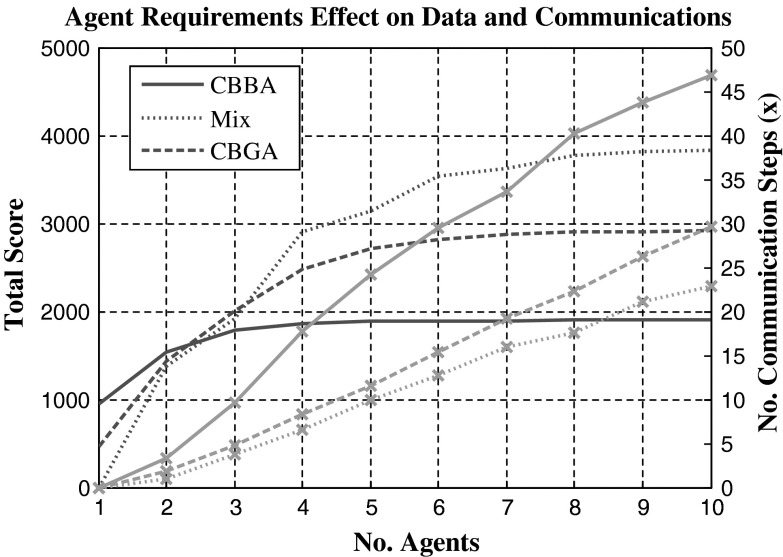
Comparison of total score and number of communication steps between the CBBA, CBGA and using both. *Markers* (×) used to show communication steps for consensus

To test the relative effectiveness of multi-agent task assignments, comparisons are drawn to that of the CBBA where each task requires a single agent. Using the CBGA, Fig. 4 shows the successful assignment of multi-agent tasks where each task requires two agents, the experiment is run in three dimensions but for easier visualisation only displayed in one dimension over time. Figure 5 has three experiments plotted that tested both algorithms, single-agent tasks use the CBBA and multi-agent tasks use the CBGA. The first experiment used just the CBBA where each task required a single agent to complete it. The second experiment tested the CBGA by requiring two agents to complete each task, and the assignments are seen in Fig. 4. The final experiment used both types of tasks making the agents consensus on assignments for 10 multi-agent tasks and 10 single-agent tasks.

In the experiment, the multi-agent tasks initially provide lower scores than the single-agent tasks but as the number of agents increases a greater increase in score is observed. Interestingly, the total number of communication steps actually decreases with the introduction of multi-agent tasks, this is significant when noted that the tasks in the experiment 2 double the total number of assignments required for consensus from 20 assignments to 40 because each task requires two agents instead of one agent.

**Fig. 6:**
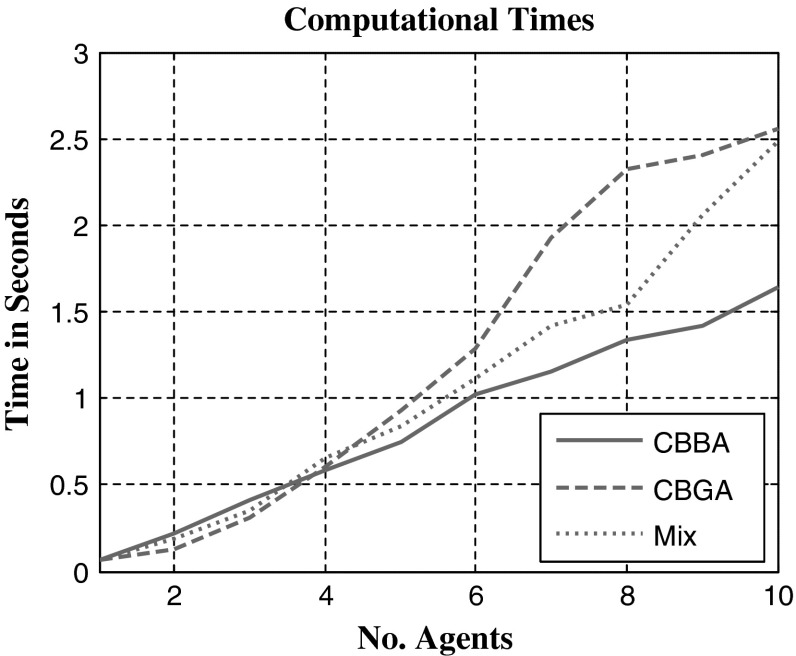
Computational times for running each experiment in Fig. 5. CBBA assigns only single-agent tasks, the CBGA assigns multi-agent tasks, and mix requires both the CBGA and CBBA

As expected, the computational times in Fig. 6 show the CBGA takes longer to come to a consensus, and this was probable due to the increased complexity of assignments. The CBBA solves single-agent assignments where each task will require 1 assignment. The CBGA solves multi-agent assignments where each task will require more than one assignment. The CBBA in Fig. 5 had to solve 20 assignments, 1 per task; alternatively, the CBGA had to solve 40 assignments because each task required 2 agents. Comparing computational time and number of communication steps, the CBGA takes a longer time to compute the consensus when receiving new assignments, but requires less overall communication between agents to achieve the final consensus.

Looking at the movement of agents in Fig. 4 presents reasons why communication drops are observed for multi-agent tasks with the CBGA. Between *t* = 0 and 60, each agent assigns and completes its initial task along with another agent. After completing the first task, agents commonly stay together for succeeding tasks, with the closest task yielding the highest reward neither agent needs to dispute the best choice. Occasionally, two groups may attempt the same task, which will then require consensus but overall each self-made group continues through the simulation effectively as one entity. With 5 agents mean communication steps decreased significantly (decrease of 14 steps) between the CBBA (24 ± 8 steps) single-agent tasks and the CBGA (10 ± 2 steps) multi-agent tasks as shown in Fig. 5. At 10 agents mean communication steps for consensus decreased further (decrease of 24 steps) showing a significant communication drop for consensus from single-agent tasks (47 ± 14 steps) to multi-agent tasks (23 ± 4 steps). Improvements are significant to *p* < 0.01 for statistical significance at 1 %.

**Fig. 7:**
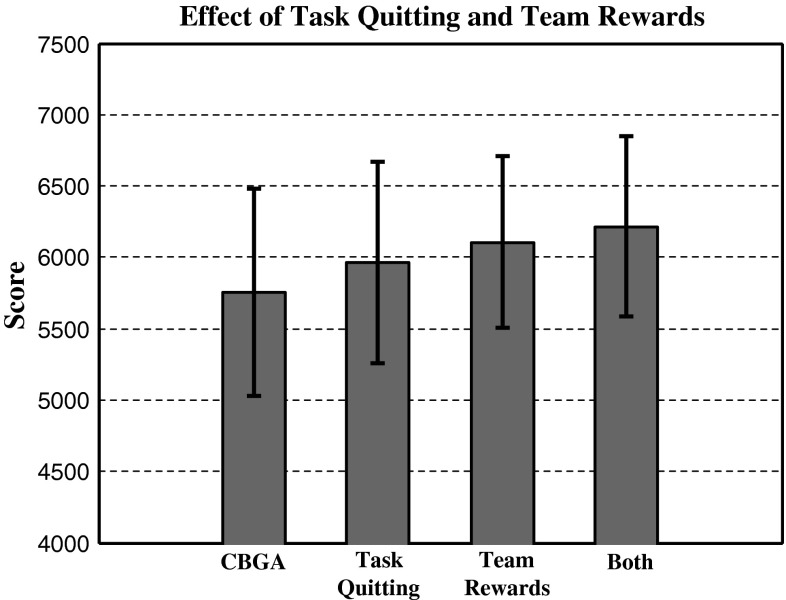
Effects of adding task quitting and team rewards to the CBGA for multi-agent tasks. Experimental score used 10 agents completing 20 tasks where each task required 4 agents for successful completion

When addressing multi-agent tasks using an algorithm that focuses on individual improvement, additional agent incentive is required to increase the effectiveness of multi-agent assignments. Task quitting and team rewards were added, and their improvements can be seen in Fig. 7. Using either task quitting or team rewards produced more complete assignments which in turn provided a higher score. Implementing task quitting on its own provides an average increase of 206 with an average score of 5,962 ± 708. Another improvement of 144 can be achieved by assigning with respect to the team rewards over task quitting producing an average score of 6,106 ± 601 but this improvement is only significant to *p* < 0.15. Further improvements are gained from using both functions increasing the mean base CBGA score from 5,756 ± 722 to a mean score of 6,216 ± 634 with a statistical significance to *p* < 0.01.

**Fig. 8:**
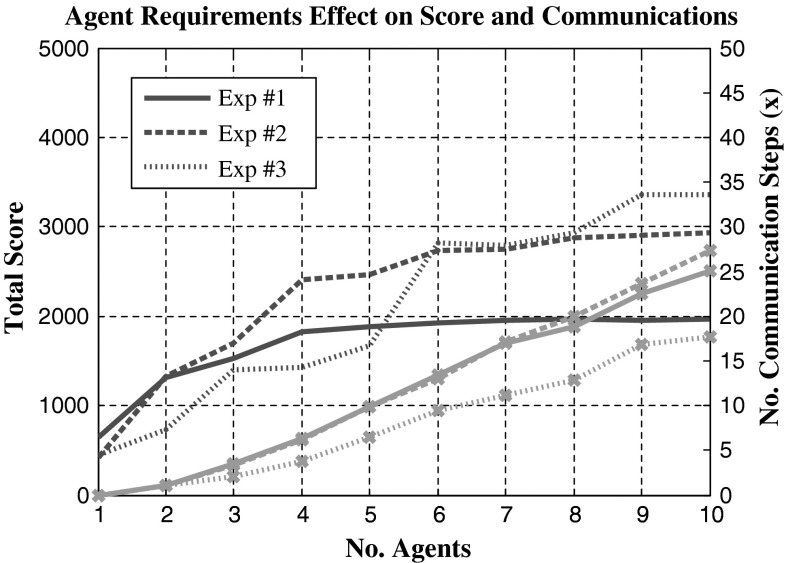
Comparison of total score and number of communication steps for tasks that require multiple different heterogeneous agents. *Markers* (×) used to show communication steps for consensus

**Fig. 9:**
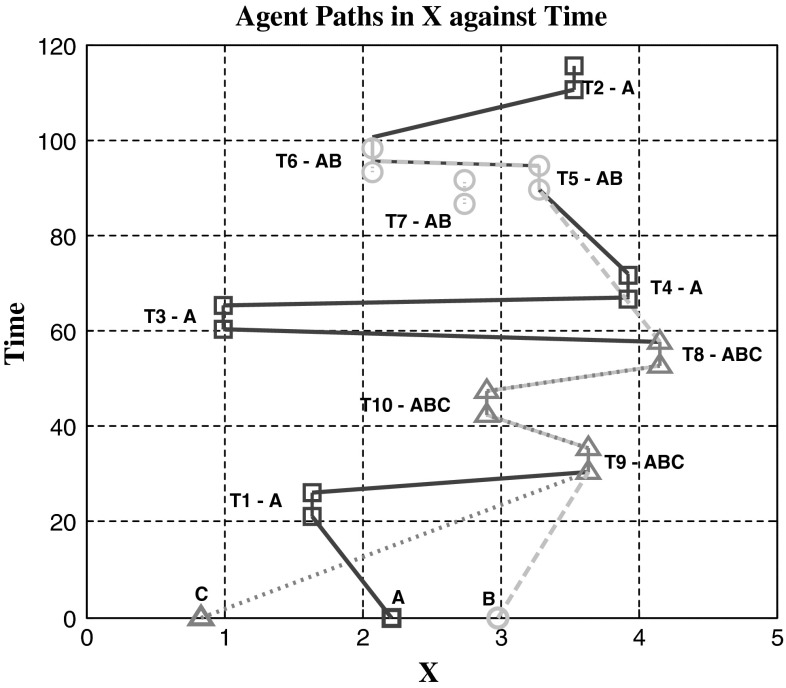
Agent’s paths through time for 3 agent types (*A*, *B* and *C*) completing 3 different types of tasks (*TA*, *TB* and *TC*)

As task complexity increases, heterogenous agents are introduced. Figure 8 shows three experiments involving heterogenous agents and tasks: experiment #1 has two agent types A and B complete solo tasks half requiring agent type A and half requiring agent B. Experiment #2 contains the same scenario as found in experiment #1 except a third type of task is added that requires both agents A and B. Finally, experiment #3 contains three types of agents and three different tasks requiring agents A, AB and ABC, respectively. Agents are split evenly between the three types with uneven splits focusing on agent A then B first. Tasks are fixed at 20 in each simulation with a split of 8–6–6 between the three tasks A, AB and ABC. Figure 9 shows a typical assignment of experiment #3 with reduced tasks for visual clarity. A reduction in the communication required to meet a consensus is observed once a task requires all three equipped agent types. These results might be a consequent of the time constraints on the tasks which will limit the available options from the maximum 20 tasks down to a much easier to manage set of the earliest obtainable. In Fig. 9, for equipment-dependant multi-agent tasks, it is seen how agent C has very little choice in its assignments and causes no conflict with other agents because it must depend on its teammates to arrive and aid its tasks. Agent A freely moves between its tasks and, when required, aids its teammates. The reduced options for each agent greatly reduce the length of communication time required between team mates. More importantly, the reduced amount of conflicts caused helps agents come to a quick consensus with smaller communication exchanges.

**Fig. 10:**
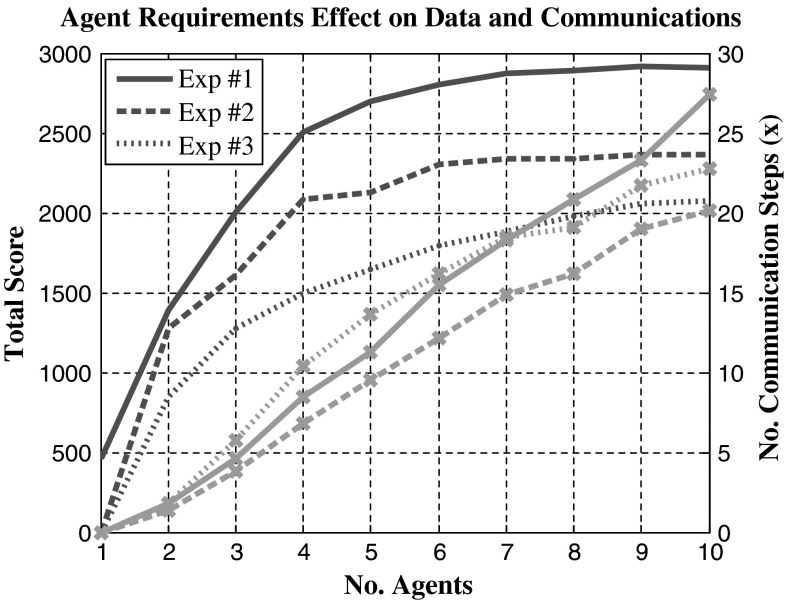
Comparison of total score and number of communication steps for tasks that require previous tasks completed. Exp #1 used multi-agent tasks with no task dependency. Exp #2 and #3 both added task dependencies but Exp #3 removed the time requirement on follow-up tasks. *Markers* (×) used to show communication steps for consensus

**Fig. 11:**
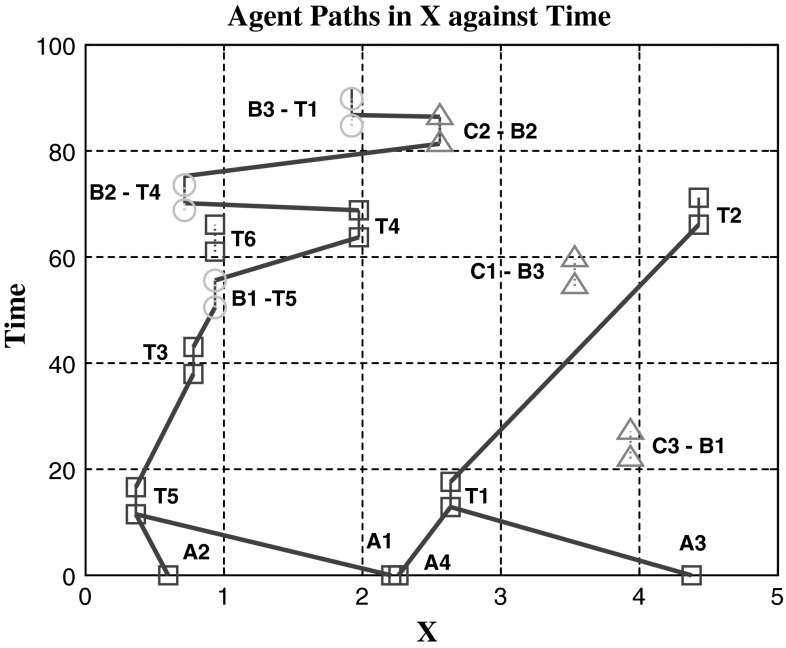
Agents (*A*) movement through time and the *X* axis. Tasks marked ‘B’ require the corresponding task ‘T’ to be completed first, similarly tasks marked ‘C’ require a task ‘B’ completed

Figures 10 and 11 show the introduction of tasks that have prerequisites where a specific task must be scheduled for completion before the task with the prerequisite requirement is assigned. It shows that compared to CBGA tasks found in experiment #1, the number of communication messages sent to reach a consensus is usually lower with the additional restrictions. By putting these prerequisite requirements on half the tasks in the simulation, we reduce the number of tasks that agents find conflict over, with the follow-up tasks having fewer conflicts. Experiment #2 contained a problem where the task time window for completion was randomly generated. In some cases, this meant a task with requirements was set before that of the requirement. This error created a number of tasks that could never be achieved and therefore limited the overall score obtainable. Interestingly, when these time limits were removed in experiment three, the average score decreased even though more tasks had become available. This might be because only agents who were involved in the prerequisite could attempt the follow-up task, but often they would be busy completing other tasks. Although in contrast after opening up accessibility on these tasks, the overall communication levels increased. When tasks are made accessible to everyone, points of conflict are amplified and therefore the number of communication steps required to come to a consensus is also increased. By limiting tasks to a small subset of agents, the overall requirements on communication for consensus decrease.

## Conclusions and Discussions

This paper presented an extension of the CBBA that solves the multi-agent multi-task assignment problem with group- and equipment-based dependencies. The new storage of data and communication in the paper enables agents to deal with multiple assignments on tasks and allows consensus in a dynamic environments between multiple heterogeneous agents. Additionally, the amount of data communicated has been reduced for multi-agent tasks. Inspiration from biological systems is used to create conflict-free multi-agent assignments. Aspects from the biological social structures in bees and ants were used to improve team-focused consensus in multi-agent assignments. Using bee inspired task quitting agents can re-assign themselves to higher-demanded tasks by removing failed team assignments where requirements are not met. Statistical results show that using these biologically inspired functions created significant improvements to the multi-agent assignment problem. A further increase in the quality of assignments is achieved with team-focused rewards as seen in Fig. 7. In addition, the use of task quitting not only improved the CBGA but its use and results showed how task quitting can redistribute resources to high-demand areas as suggested by its existence in bee colonies. The process of evolution has created species of animals, such as eusocial insects, that show self-organising and adaptive qualities that can be observed and exploited to improve bio-inspired robot and agent systems.

As expected, the increased complexity of the multi-agent problem compared with the single-agent problem has increased the computational time for consensus. However, the computational time is similar for smaller groups of agents shown in Fig. 6. Contrary to what was expected the number of communication steps required for consensus has decreased for both single- and multi-agent tasks. Observation of agent paths in Fig. 4 suggests that after the initial assignment, agents are likely to stay together in groups that potentially provide quick consensus with little conflict. With agents staying together for assignments, the effective number of agents with conflicting bids is reduced, thus the number of communication steps for consensus is lower.

For multi-agent problems, agents group up to complete tasks and in some cases for the entire simulation. With increasingly, complicated group and equipment requirements groups are found to continue working together where possible, but often an agent will leave to complete another task and merge back again in a later task. By restricting tasks to a smaller subset of agents with together task requirements, the number of conflicting assignments is reduced. When cooperation is a forced requirement, it in fact simplifies the problem rather than completes it.
